# An evaluation of barriers and facilitators to implementing multiplex rapid antigen testing for SARS-CoV-2 and influenza A and B in congregate living settings

**DOI:** 10.3389/fpubh.2025.1560131

**Published:** 2025-04-07

**Authors:** Yasmin Garad, Andreea A. Manea, Negin Pak, Lames Danok, Stefan Baral, Tom Dykstra, Danielle Kasperavicius, Sharon E. Straus, Christine Fahim

**Affiliations:** ^1^Knowledge Translation Program, St. Michael’s Hospital, Unity Health Toronto, Toronto, ON, Canada; ^2^Department of Epidemiology, Johns Hopkins School of Public Health, Baltimore, MD, United States; ^3^Toronto Shelter and Support Services, City of Toronto, Toronto, ON, Canada; ^4^Department of Medicine, University of Toronto, Toronto, ON, Canada; ^5^Institute of Health Policy, Management and Evaluation, University of Toronto, Toronto, ON, Canada

**Keywords:** SARS-CoV-2, influenza A and B, rapid antigen testing, congregate living settings, implementation strategies, barriers and facilitators

## Abstract

**Introduction:**

Point of care multiplex rapid antigen testing (RAT) is a tool that can be used to mitigate and respond to facility-based infectious disease outbreaks. However, little is known about how to optimally implement this testing in congregate living settings (CLSs), including long term care homes (LTCHs), retirement homes (RHs), and shelters serving people experiencing homelessness. Our objective was to explore the barriers and facilitators to implementing a new device for multiplex RAT for COVID-19 and influenza across CLSs in the Greater Toronto Area, Canada.

**Materials and methods:**

Using key informant interviews, we assessed barriers and facilitators to implementing multiplex RAT across CLSs. Qualitative coding using the framework approach was used to identify themes. We used the Theoretical Domains Framework (TDF) and the Consolidated Framework for Implementation Research (CFIR) to identify individual and contextual-level barriers and facilitators to implementation. Identified barriers were then mapped to implementation strategies using theoretically-rooted frameworks and tools.

**Results:**

We completed 45 interviews with staff at CLSs (8 LTCHs, 4 RHs, 12 shelters) between January 2022 and March 2023. Four barriers to RAT implementation in CLSs emerged including: limited material resources for implementation; insufficient staff capacity to perform RAT testing; complexity of RAT implementation; and reluctance among staff to adopt a new testing process. Five facilitators to implementation were described including: training and implementation support for staff at the CLSs; site-level implementation champions; access to materials to support testing; perceived advantages of simultaneous testing for COVID-19 and influenza; and the usability and functionality of the RAT testing device. Twenty implementation strategies were identified through implementation strategy mapping.

**Discussion:**

Multiplex RAT options can empower CLS staff to promptly identify and respond to viral respiratory outbreaks. The use of evidence-based implementation strategies can enhance the effectiveness of using multiplex RAT to control outbreaks in CLSs.

## Introduction

Congregate living settings (CLSs) including long-term care homes (LTCHs), retirement homes (RHs) and shelters serving people experiencing homelessness represent high-risk settings for SARS-CoV-2 transmission in Canada (1–3). Crowding, frailty and pre-existing medical conditions put residents of CLSs at higher risk of COVID-19 and influenza infection and death, compared to the general population (4, 5). Despite relatively low influenza burden in 2020–2021 in Canada, concerns were raised that CLSs would experience a resurgence of influenza cases in subsequent years, alongside COVID-19 (6). Reasons for this concern included limited recent infection-derived influenza immunity due to large scale public health interventions adopted during the COVID-19 pandemic (6, 7). This concern was realized when influenza rose to epidemic levels in Canada between April 2022–June 2022 (8). CLSs have historically been prone to influenza outbreaks, which highlights the importance of multiplex rapid antigen testing (RAT) as a critical tool for managing both influenza and COVID-19 (9).

Delayed implementation of screening and infection prevention and control (IPAC) measures for SARS-CoV-2 in CLSs in Ontario posed challenges to early detection and management of outbreaks during the COVID-19 pandemic (1). Further, influenza and COVID-19 symptoms are similar, but IPAC strategies vary. For instance, there is more emphasis on social distancing/quarantining for COVID-19 (e.g., limiting visitors, mask mandates, remote services and telehealth, isolation protocols, etc.) because COVID-19 spreads more easily than influenza (10, 11) and can lead to more severe illness and complications for some individuals (12). Thus, the ability to discern infections in real time is critical to implementing timely programs and policies to mitigate outbreaks (13) and enabling targeted treatments for COVID-19 and influenza. Multiplex testing simplifies operations by identifying multiple pathogens from a single sample, supporting tailored responses (14).

A multiplex RAT platform for both COVID-19 and influenza A and B offers a practical tool for infection prevention and control in CLSs by efficiently providing multiple results from a single sample. Multiplex RAT may enable early identification of respiratory infections in residents and staff and allow for the timely application of outbreak prevention strategies (e.g., isolation of affected individuals) (13, 15). However, little is known about how to optimally implement, scale up, and sustain these tests in CLSs (16–18).

We aimed to explore the barriers and facilitators of multiplex RAT for COVID-19 and influenza using test kits from Quidel Corporation, San Diego, California, along with their Sofia® 2 Analyzer across several Ontario CLSs. In a recent systematic review that assessed 49 different types of RATs for COVID-19, the Sofia^®^ Flu + SARS Antigen Fluorescent Immunoassay (FIA) (hereafter Sofia RAT) was one of seven tests that met the World Health Organization’s performance standards for sensitivity, and one of twelve that met these standards for specificity (19). Implementation of multiplex RAT for COVID-19 and influenza using the Sofia RAT took place between December 2021 and March 2023 as part of an initiative to improve IPAC practices across CLSs in the Greater Toronto Area, Ontario, Canada. Once barriers and facilitators were identified, they were mapped to corresponding, theoretically rooted implementation strategies that could be used to support and sustain multiplex RAT implementation in CLSs.

## Materials and methods

### Study design

We conducted a qualitative study using the framework approach (20) to assess the perceived barriers and facilitators of implementing the Sofia RAT with the Sofia 2 Analyzer across CLSs in the Greater Toronto Area, Ontario, Canada (population 6,372,000). The Sofia RAT uses immunofluorescence technology to simultaneously detect nucleocapsid protein from influenza A, influenza B and SARS-CoV-2.It is paired with the Sofia 2 Analyzer, which is a small bench top analyzer that uses an ultraviolet LED energy source to automatically read each test in 15 minutes (21, 22). The Sofia 2 Analyzer also allows for the electronic storage of data on test results, which can be exported.

Retirement homes, shelters, and long-term care homes in the Greater Toronto Area that had the highest resident capacity were prioritized for study recruitment. Due to the limited number of analyzers available for implementation, the maximum number of LTCHs and RHs that could participate simultaneously was twelve. With the support of a mobile health team, implementation across a larger number of shelters was possible. Implementation took place across six RHs, twelve shelters and seven LTCHs, in the Greater Toronto Area. Testing was open to staff but was focused mainly on residents, as staff who were symptomatic or were exposed to COVID-19 typically completed self-testing at home.

#### Theoretical framework

Data collection and analysis for this study were guided by the 2022 Consolidated Framework for Implementation Research (CFIR) (23–25) and the Theoretical Domains Framework (TDF) (26, 27). The CFIR is a meta**-**framework that is used to guide the identification of factors that may influence the implementation and effectiveness of an intervention (23, 24). It includes several constructs that have been associated with effective implementation, organized across five domains. Similarly, the TDF is a framework used to assess factors that influence individual behavior change (28). The TDF can be used alongside the Capability, Opportunity, Motivation-Behavior (COM-B) model, a theory that suggests that behavior is impacted by an individual’s capabilities, opportunities and motivations to change; these factors can interact with contextual factors to determine uptake of interventions (29). We used the TDF and the CFIR to identify individual-level and contextual-level barriers and facilitators to change, respectively. We then used implementation tools to identify theoretically-linked strategies that could be used to mitigate barriers and leverage facilitators to change (30, 31).

#### Participant selection

Purposive and snowball sampling were used to recruit interview participants across the 25 CLSs participating in the multiplex RAT implementation project (32, 33). Purposive sampling ensured the inclusion of individuals directly involved in implementation, such as site leadership and frontline staff, to capture diverse perspectives on adoption, sustainability, and scale-up. Efforts were also made to recruit participants from CLSs at different stages of implementation. Snowball sampling further expanded recruitment by identifying additional staff with relevant experience who may not have been initially identified.

Individuals involved in implementation at these CLSs were identified through leadership at each site and invited to participate in this study. All individuals identified were then contacted via email for interview recruitment (*n* = 57). Once potential participants confirmed interest, an interview was scheduled and informed consent was obtained. Participants were compensated using $20 CAD gift cards upon study completion.

#### Setting

The population of Ontario, Canada is 15.8 million people (34). There are approximately 1439 RH, 157 shelters and 627 LTCHs in the province, of which 64.5, 37.4 and 33.8%, respectively, are found in the Greater Toronto Area (35–40). Multiplex RAT implementation across the 25 participating CLSs was launched on a rolling basis between December 2021 and March 2023 (representing the COVID-19 Omicron variant waves in the province) (41).

#### Data collection

Semi-structured interview guides were rooted in the CFIR (23–25) and the TDF (26, 27) (see Appendix A). To capture barriers and facilitators throughout the implementation period, we conducted interviews at different stages of implementation. Interviews conducted at earlier stages of implementation examined existing workflows and identified barriers and facilitators to adoption, informing the co-creation of an implementation strategy with CLS staff. Interviews conducted at later stages of implementation, explored factors influencing the sustainability and scale-up of rapid testing in CLSs. Some individuals participated in both baseline and follow-up interviews to provide insights into changes over time. Interviews were conducted with staff at CLSs at various stages of implementation, ranging from 1 to 15 months post-implementation.

Interviews were conducted remotely over the phone or through a secure videoconferencing software (Zoom; audio setting only) by members of the research team [YG, OO, MB, AM]. Interviews lasted 30 to 60 min and were offered in English. Only interviewees and researchers were present at the interviews. The interviews were audiotaped and then transcribed verbatim using NVivo 12 (42). The research team [AM, MB, NP, LD] de-identified the transcripts and reviewed them for accuracy.

Demographic data were collected using an electronic survey that was circulated via email after the interviews (see Appendix B). In addition to demographic characteristics (e.g., age, gender), participants were asked about the type of CLS they worked at (LTCH, RH, or shelter), their role, and their level of involvement in the implementation of RAT using the Sofia 2 Analyzer (see Appendix B).

### Analysis and findings

In keeping with the framework method (20), both inductive and deductive coding were used to categorize individual and contextual implementation barriers and facilitators (23–25, 28). Research staff (NP, LD) developed a codebook guided by the frameworks. Coding was conducted using NVivo 12 (42). Research staff double coded a 10% sample of transcripts until a kappa of 0.6 (moderate) agreement was reached, after which the remaining transcripts were independently coded (NP), and reviewed by a second reviewer (LD). The data were coded to identify emergent themes, and the codebook was iteratively revised as needed to accommodate additional themes (NP, LD). Themes were then categorized using the CFIR and the TDF domains by one coder (YG). Differences over time were assessed by comparing interview responses related to each theme at different point in implementation.

To identify corresponding implementation strategies, one coder (YG) mapped CFIR domains using the CFIR-Expert Recommendations for Implementing Change (ERIC) Barrier Buster tool and TDF domains were mapped to strategies identified using the SELECT tool (30, 31). The CFIR Barrier Buster tool generates a list of ERIC implementation strategies, prioritized by highest to lowest cumulative percentage of endorsement across all selected CFIR barriers. Implementation strategies with a cumulative percentage greater than 50% were chosen and combined with implementation strategies generated through the SELECT tool, which leverages Michie’s COM-B model to map TDF domains to COM-B intervention functions and intervention strategies corresponding to these functions.

Demographic data were analyzed using descriptive frequencies by a research coordinator (YG). We report our methods and findings in accordance with the consolidated criteria for reporting qualitative studies (COREQ, Supplementary file 1) (43). This study was approved by the St. Michael’s Hospital evaluation/quality improvement project approval process, ReQuIST (Review of Quality Improvement Studies) (ReQuIST Number: 166).

#### Research team and reflexivity

Interviews were conducted by research staff from the Knowledge Translation Program at St. Michael’s Hospital in Toronto, Ontario, Canada (YG, OO, MB, AM). Interviewers held a Bachelor’s or Master’s level degree and were experienced in qualitative methodology. All interviewers were women of diverse racial backgrounds who worked as research assistants or research coordinators. An interview guide with predefined questions and prompts was developed to reduce the risk of bias while interviewing and during analysis (see Appendix A). Additionally, the research staff engaged in self-reflection exercises to recognize and mitigate the risk of bias (44). Research staff had previously established relationships with some of the interview participants through collaboration on research projects. While these pre-existing relationships facilitated trust, they may have influenced participant responses and interactions during the interview process.

## Results

Twenty-four out of 25 intervention sites (96%) agreed to participate in this study. A total of 57 individuals were invited for interviews, of whom 39 participated (response rate = 68%). These participants collectively participated in 46 interviews conducted between January 12 2022 and March 31 2023. The remaining 18 individuals either left their organization, were unavailable due to competing priorities, or did not respond to scheduling attempts.

### Demographic characteristics

Participants represented a range of ages, professions, and other demographic characteristics. Ten percent of participants worked in LTCHs, 41% worked in RHs and 49% worked in shelter settings (see Table 1). The majority of interview participants were women (*n* = 24, 61.5%) and college/university educated (*n* = 34, 87.2%; see Table 1). Participants were evenly distributed between age groups: 15.4% (*n* = 6) were aged 18–30, 20.5% (*n* = 8) were aged 31–40, 28.2% (*n* = 11) were aged 41–50 and 17.9% (*n* = 7) were over the age of 50. The sample included individuals from diverse racial backgrounds, with approximately half identifying as racialized (*n* = 19, 48.7%). Individuals who identified as Black/LatinX represented 15% of the participant population (*n* = 6), and 33.3% (*n* = 13) of participants identified as South Asian, East Asian or Southeast Asian. Additionally, 51.3% were foreign-born Canadian citizens or Permanent Residents (*n* = 20). Participants held a wide range of roles, including nurses, IPAC practitioners, directors, managers, quality improvement staff, personal support workers and physicians. Most participants worked full time (*n* = 29, 74.4%) and had been working in their role for at least 4 years (*n* = 17, 43.6%). Most respondents reported that they were moderately or highly involved in RAT implementation at their site (*n* = 24, 61.5%).

**Table 1:** Demographic characteristics.

	Overall (*n* = 39)	
	n	%
Age (years)		
18–30	6	15.4%
31–40	8	20.5%
41–50	11	28.2%
>50	7	17.9%
Not reported/prefer not to answer	7	17.9%
Gender		
Man	8	20.5%
Woman	24	61.5%
Not reported/prefer not to answer	7	17.9%
Race/Racial Identity		
Black/Latinx	6	15.4%
South Asian/East Asian	5	12.8%
Southeast Asian	8	20.5%
White	11	28.2%
Other/not reported	9	23.1%
Immigration Status		
Canadian citizen (born in Canada)	13	33.3%
Canadian citizen (foreign born)/Permanent resident	20	51.3%
Not reported/Prefer not to answer	6	15.4%
Highest Level of Education		
College/University Degree	27	69.2%
Post-graduate Degree	7	17.9%
Other/not reported	5	12.8%
Site Type		
LTCH/RH	20	51.3%
Shelter	19	48.7%
Role		
Director	8	20.5%
IPAC Practitioner	5	12.8%
Manager	7	17.9%
Clinician (Physician or Nurse)	11	28.2%
Not reported/other	8	20.5%
Employment Status		
Full-time	29	74.4%
Part-time/not reported	10	25.6%
Number of Years in Role		
<1–3 years	12	30.8%
4–6 years	10	25.6%
> 6 years	7	17.9%
Not reported	10	25.6%
Level of involvement		
Limited involvement	5	12.8%
Moderate involvement	11	28.2%
High level of involvement	13	33.3%
Not reported	10	25.6%

### Barriers and facilitators to multiplex rapid antigen testing implementation

We observed four main barriers and five facilitators to RAT implementation in the CLSs. Barriers and facilitators identified at baseline continued to persist for the study period (see Tables 2, 3 for summary of themes and participant quotes). Barriers to implementation included limited material resources for implementation (e.g., more devices were needed at some sites due to a high volume of residents), lack of staff capacity (e.g., staff shortage, lack of trained staff), complexity of implementation, reluctance among staff to adopt a new testing process, and preferred alternatives to the Sofia RAT (see Table 2).

**Table 2:** Barriers to multiplex RAT implementation.

Theme	Domain (Framework)	Construct (Framework)	Quotes	Variation over time
Limited material resources for implementation (e.g., more devices were needed at some sites due to a high volume of residents)	Inner Setting (CFIR), Environmental context and resources (Environmental constraints) (TDF)	Available resources: materials and equipment (CFIR), Resources/ material resources (availability and management) (TDF)	“Our antigen rapid testing Sofia machine is located on the second floor so sometimes it creates challenges taking some clients who could be older or fragile just to test them there”—P10, Support Worker “You have, you know, issues to deal with—the emergencies and stuff. So, for us, and it being the only machine in the building. So, I’m coming over here, and then somebody is calling me over there sometimes, it can be quite chaotic.”—P39, IPAC Practitioner	Minor variations observed. More interviewees identified this as a barrier post implementation.
Lack of staff capacity (e.g., staff shortage, lack of trained staff)	Inner Setting (CFIR), Environmental context and resources (Environmental constraints) (TDF)	Work infrastructure (CFIR), Environmental stressors (TDF)	“More staff need to be trained in order to conduct our process more smoothly, because sometimes it’s only one person if I’m the shift leader, and I have to deal with some situation and then I have to do the testing for the client that that’s very challenging”—P10, Support Worker “I think the challenges have been the staff are exquisitely overworked. And, you know, it’s like even though when we tell them about this new intervention, people are excited about it, but then like actually getting them to do the testing, etc., is like an uphill battle …because they are just so busy.”—P19, Clinician	None observed. Lack of staff capacity was reported as a barrier thoughout the implementation period.
Complexity of implementation	Innovation (CFIR)	Innovation complexity (CFIR)	“They prefer using the strip test, it’s less risky…it’s kind of like, you know, it’s just a strip, what could go wrong?”—P02, Clinician “That’s the part that scares me a bit is that it’s electric…. it needed that special calibration code.”—P22, Manager “I think the downsides are it needs to be calibrated, so staying on top of that.”—P28, Director	None observed. The complexity/technical nature of the process was a perceived barrier throughout the implementation period.
Reluctance among staff to adopt a new testing process due to a lack of familiarity, lack of capacity, cost, complexity, lack of autonomy.	Characteristics (CFIR), Inner Setting (CFIR), Knowledge (TDF)	Motivation (CFIR), Capability (CFIR), Compatibility (CFIR), Procedural Knowledge (TDF)	“I would say, like three of the sites mentioned the swabs being too big right for just rapid antigen testing, I’m assuming, because they have gotten used to smaller swabs with rapid antigen testing.”—P30, IPAC Practitioner “Some of the nurses or staff are older…. the machine confuses, frightens them, it’s expensive, so they are less willing to use it”—P02, Clinician	None observed. Staff reluctance due to several factors was reported by interviewees throughout the implementation period.

**Table 3:** Facilitators to multiplex rapid antigen testing implementation.

Theme	Domain (Framework)	Framework (Framework)	Quotes	Variation over time
Online and in person training and implementation support for staff at the CLSs	Readiness for Implementation (CFIR), Knowledge (TDF)	Access to Knowledge & Information (CFIR), Procedural Knowledge (TDF)	“Being available even if it’s like a small question, giving reassurance, and then training, that helps with barrier relating to discomfort of using a machine rather than calling helpline.”—P02, Clinician “I think the education to begin with was done well. We knew what to do. We had practiced through the education, so that was done well and would have been able to roll out quite easily.”—P31, Manager	None observed. Training was provided prior to implementation and on an ongoing basis as needed after intervention launch.
Identifying implementation champions at the site level	Individuals (CFIR), Social influences (Norms) (TDF)	Implementation Leads (CFIR), Mid-level leaders, Champions (TDF)	“Having specific health care staff that that are specifically trained in doing that, who do not have another job on the side [would be helpful to implementation]”—P11, Clinician “I had a lot of support. I’ve had support from my infection control coordinator, right? So she was able to assist me. And also, just looking through the menus, I had another staff member who was familiar with the Sofia 2 analyzer, who was able to help me with that. So, it worked out well.”—P32, Support Worker	None observed. Implementation champions were an anticipated facilitator prior to implementation. Post implementation, champions were noted as an anticipated facilitator by some participants and actual facilitator by others.
Access to materials to support testing (e.g., test kits, calibration cassettes, timers)	Inner Setting (CFIR), Environmental context and resources (Environmental constraints) (TDF)	Available resources: materials and equipment (CFIR), Resources/ material resources (availability and management) (TDF)	“Being available even if it’s like a small question, giving reassurance, and then training, that helps with barriers relating to discomfort of using a machine rather than calling helpline.—P02, Clinician “In terms of supplies, it’s been accessible. If there is more than one client, we can still perform the test.”—P38, Clinician	Minor variations observed. Access to materials was an anticipated facilitator prior to implementation. Post implementation, more interviewees identified this as a barrier.
The ability to test for both COVID-19 and influenza concurrently	Inner Setting (CFIR)	Tension for Change (CFIR)	“(If) program is flexible, we are looking at alternative ways this machine can be used, it’s very sustainable. It could be used in intakes, or it could be used during flu season, which comes around every year.”—P02, Clinician “[Shelter staff] mentioned how convenient it is, how beneficial it is to have an analyzer machine that is able to differentiate between the flu and the COVID-19 so that they know how to isolate or not based off those people’s symptoms just because the symptoms do mimic one another.”—P30, IPAC Practitioner	None observed. The value of being able to test for both COVID-19 and influenza was seen as a facilitator throughout the implementation period.
Functionality of the device (e.g., ease of interpretation of results, simplicity, mobility, quick results with the “read now” option)	Inner Setting (CFIR)	Tension for Change (CFIR)	“I like the ability that it records. I like to believe that I can walk away from it, and it’ll still display.”—P22, Manager “It was pretty easy, and it was amazing how quickly you got results instead of waiting that 15 min like we have to for like our normal rapid tests that we have.”—P35, Clinician	None observed. Participants interviewed prior to implementation anticipated that functionality of the device would be a facilitator. Participants also noted this as a facilitator post-implementation.

Facilitators to implementation included online and in-person training and implementation support for staff at the CLSs, implementation champions at the site level, access to materials to support testing, and the benefit of being able to test for both COVID-19 and influenza. CLS staff also found the device to be very functional (see Table 3). Several participants expressed that their site wanted to continue to use the multiplex Sofia RAT beyond the scope of the project, due to its ability to detect multiple pathogens.

#### Barrier—limited material resources for implementation

Limited access to material resources was a barrier expressed by several participants. Each site that participated in implementation was provided with one Sofia 2 Analyzer. For sites that were large and/or had multiple floors, having access to only one analyzer created longer testing wait times. It also created a barrier for residents with mobility challenges. Although the analyzer was mobile, having access to only one meant that the machine was often assigned to a specific location at large CLSs. This increased the burden for site staff that attempted to test patients in one location while managing care needs in other areas of the building.

#### Barrier—lack of staff capacity

Many interview participants noted a lack of staff capacity as a barrier to the implementation of the multiplex Sofia RAT. Participants noted that staff were overworked and that some sites had high rates of staff turnover. During the implementation period, some sites decided to train only a few of their staff to use the Sophia RAT. This led to a barrier when the trained staff members were not on site or were busy with other tasks. Also, some trained staff felt that their workloads had increased as a result of the RAT testing.

#### Barrier—complexity of implementation

Many interview participants mentioned that they found the administration of multiplex RAT using the Sofia 2 Analyzer to be technical and complex. Some were uncomfortable with the use of the technology, while others felt that the need to calibrate the machine every 30 days created a barrier that other rapid testing options (e.g., the BTNX Rapid Response^®^ COVID-19 Antigen Rapid Test) did not pose. Additionally, the calibration process required the insertion of a small cassette that was often misplaced, which impacted staff’s ability to calibrate the machine.

#### Barrier—reluctance among staff to adopt a new testing process

Factors that influenced reluctance among staff to adopt this new testing process included the complexity of the machine as well as the swab size. Some participants noted that the swabs were thicker than what they were accustomed to from other rapid tests, which was uncomfortable for residents. Additionally, the complexity of the testing process acted as a deterrent to continuing the use of the Sofia RAT despite initial interest.

#### Facilitator—online and in person training and implementation support for staff at the CLSs

Training resources in the form of modules and demonstrations (online and in-person) were provided to site staff. The implementation team (YG, AM, OO) was also available to respond to questions or provide onsite support as needed. Many participants found these resources to be beneficial to implementation.

#### Facilitator—identifying implementation champions at the site level

Some interview participants expressed that having an individual trained and tasked to perform multiplex Sofia RAT testing was beneficial. Similarly, having a well-trained individual who was able to assist others onsite to use the Sofia 2 analyzer helped to facilitate implementation.

#### Facilitator—access to resources to support testing

Some sites felt that they had enough materials to implement the multiplex Sofia RAT while others had higher levels of need, including the need for more analyzers to carry out testing efficiently when testing residents on multiple floors. Shelters more commonly reported a need for more resources. Despite a preference for more materials to allow for optimal implementation (e.g., shorter wait times), shelters were able to continue with testing. Retirement homes typically did not report the need for more resources. In addition to resources for testing, the availability of space to conduct testing and access to implementation were seen as facilitators for these staff.

#### Facilitator—the ability to concurrently test for both COVID-19 and influenza

Many participants expressed that the ability to concurrently test for both COVID-19 and influenza facilitated implementation due to the need to differentiate between respiratory illnesses in CLSs. This guided use of most appropriate IPAC measures and mitigated the risk of COVID-19 or influenza spread. Some participants believed that the the Sofia RAT would be especially useful during annual influenza seasons.

#### Facilitator—functionality of the device

Some staff found the analyzer to be user friendly and easy to interpret. Additionally, some individuals reported that RAT using the Sofia 2 Analyzer led to quick results (due to the “read now” mode), and its ability to keep an electronic record of all test results was also seen as beneficial.

### Strategies for future implementation

Barriers and facilitators were mapped to CFIR and TDF constructs (see Tables 2, 3). These constructs were mapped to 34 ERIC implementation strategies using the CFIR-ERIC Barrier Buster tool and 19 implementation strategies identified across 5 intervention functions (education, training, persuasion, enablement, environmental restructuring) using the SELECT tool. Duplicate strategies identified via both the CFIR-ERIC and the SELECT tool were merged, resulting in 44 unique strategies. These implementation strategies were then further refined based priorities and recommendations for strategies, as identified through interviews. This resulted in 20 identified implementation strategies (see Table 4).

**Table 4:** Intervention strategies mapped to identified barriers.

Intervention functions	Intervention strategy	Mapping tool	Implementation strategy description
Education	Conduct educational meetings	SELECT + ERIC	Hold meetings involving program targets (e.g., providers, administrators, other organizational parties, and community, patient/consumer, and family members) to improve knowledge about the ideal practice
	Develop educational materials	ERIC	Develop and format manuals, toolkits, and other supporting materials in ways that make it easier to learn about and deliver the innovation
	Distribute educational materials	SELECT	Distribute educational materials (e.g., guidelines, manuals, and toolkits) in person, by mail, and/or electronically to improve knowledge about the ideal practice
Enablement	Conduct local needs assessment	ERIC	Collect and analyze data related to the need for the innovation
	Identify early adopters	ERIC	Identify early adopters at the local site to learn from their experiences with the practice innovation
	Inform local opinion leaders	ERIC	Inform providers identified by colleagues as opinion leaders or “educationally influential” about the clinical innovation and support them to educate and influence colleagues to support uptake
	Public funding and contracting	SELECT + ERIC	Set system priorities to encourage implementation of the ideal practice by establishing government/service payer funding formulas, proposal requests and contracting for the ideal practice
	Tailor strategies	ERIC	Tailor the implementation strategies to address barriers and leverage facilitators that were identified through earlier data collection
	Use a learning collaborative/CoP*	SELECT + ERIC	Facilitate the formation of groups of providers or provider organizations, and foster a collaborative learning environment to improve implementation of the ideal practice
	Use of champions	SELECT + ERIC	Identify and prepare individuals to dedicate themselves to supporting, marketing and overcoming indifference or resistance related to implementing the ideal practice
	Conduct local consensus discussions	ERIC	Include local providers and other interested parties in discussions that address whether the chosen problem is important and whether the clinical innovation to address it is appropriate
	Provide local technical assistance	ERIC	Develop and use a system to deliver technical assistance focused on implementation issues using local personnel
Environmental restructuring	Alter incentive/allowance structures	ERIC	Work to incentivize the adoption and implementation of the clinical innovation
	Change physical structure and equipment	ERIC	Evaluate current configurations and adapt, as needed, the physical structure and/or equipment (e.g., changing the layout of a room, adding equipment) to best accommodate the targeted innovation
	Reminders	SELECT	Develop reminder systems to help providers recall information and/or prompt the performance of the ideal practice
	Revise professional roles	SELECT	Shift and revise roles among professionals who provide care, and redesign job characteristics to promote uptake of the ideal practice
Training	Conduct educational outreach visits	SELECT	Have a trained person (external to the setting/organization) meet with providers in their practice settings and educate them on how to perform the ideal practice
	Conduct ongoing training	ERIC	Plan for and conduct training for the clinical innovation in an ongoing way
	Recruit, designate and train for leadership	ERIC	Recruit, designate, and train leaders for the change effort
	Use train-the-trainer strategies	SELECT	Train designated providers or organizations so that they can train others in the ideal practice

*CoP, Community of Practice.

## Discussion

We identified four barriers and five facilitators to implementation of the multiplex Sofia RAT for COVID-19 and influenza across 25 CLSs. Individual-level barriers included a lack of staff capacity and materials needed for testing, and complexity of the testing process. System-level barriers included limited material resources. Implementation facilitators included provider training, the assignment of implementation champions, the ability to test for both COVID-19 and influenza simultaneously, and functionality of the test. Participants in our study also noted the benefit of quick results, which enabled CLSs staff to administer timely treatment and implement IPAC measures promptly to mitigate the spread of disease (45).

Our identified barriers are similar to previous studies that assessed the implementation of RAT for infectious diseases in other settings (46–51). Notably, our study assessed one specific test, the Sofia 2 Analyzer, which was perceived to be user-friendly. It is important that other tests be evaluated for usability, given that ease of use is an important contributor to uptake and implementation of RAT testing (52–57). Our participants also noted the importance of dual point care testing, which has been found to save time, reduce patient burden, and lead to cost-savings (50, 58–60). Additionally, the ability to conduct testing onsite for multiple infectious diseases reduces obstacles experienced by individuals living in CLSs, such as access challenges due to a lack of mobility or limited funds (e.g., inability to travel to healthcare facilities) (53, 61, 62).

There is a paucity in the literature regarding solutions to identified barriers in the context of rapid antigen testing implementation. However, there is some evidence that suggests that workforce capacity can be improved by automating some administrative tasks (e.g., documenting results), training programs for staff, modifying procedures and task shifting (63–66). These adjustments can also reduce staff burnout which can in turn enhance staff capacity (63, 67). Research also indicates that staff training and integrating the implementation of complex tools into existing workflows can support their successful adoption in healthcare settings (68). Lastly, to mitigate the risk of limited material resources, policy interventions, including digital infrastructure to track the utilization of products, strategic stockpiling, and supply stewardship when needed, may be beneficial (69–71).

We mapped our findings using theoretically-rooted frameworks to identify 20 implementation strategies that can be considered to mitigate barriers and leverage facilitators for the routine implementation of the Sofia 2 Analyzer in CLSs. CLSs considering RAT implementation should further refine this list of strategies, based on their perceived barriers, facilitators and context, and should consider elements of feasibility, acceptability, and cost in their selection (72).

## Limitations

Limited staff capacity in the context of the COVID-19 pandemic made it challenging to obtain a representative sample of interviews from each site involved in this study, thus potentially introducing a sampling bias in our findings. Additionally, it is possible that barriers and facilitators evolved over time, in particular as pandemic pressures eased; resource constraints limited our ability to conduct multiple interviews over a prolonged period to assess such temporal changes. Finally, we did not interview CLS residents due to access limitations related to IPAC measures, and may have missed important considerations from this perspective.

This study was conducted in the Greater Toronto Area, where CLSs operate within a publicly funded healthcare system and serve a high-density population. These conditions may not be generalizable to regions with different population demographics, healthcare funding models, mandates, or cultural attitudes toward rapid testing. Future research should examine barriers and facilitators to the implementation of point-of-care multiplex rapid testing in rural areas, different healthcare system infrastructures, and diverse geographical and cultural contexts to assess the transferability of these findings.

The use of site leadership to identify interviewees may have also introduced sampling bias. Additionally, pre-existing professional relationships between some research staff and participants may have influenced responses due to familiarity or social desirability bias. Self-reflection exercises were conducted to mitigate this risk and trust and relationship building were central to gaining access to these CLSs to facilitate this study.

## Conclusion

Multiplex RAT options can empower CLSs to promptly identify and respond to viral respiratory outbreaks. Implementing evidence-based strategies can further improve the effectiveness of RAT in controlling and responding to outbreaks.

## Data Availability

The raw data supporting the conclusions of this article will be made available by the authors, without undue reservation.
