# Supplementary material for: An evaluation of barriers and facilitators to implementing multiplex rapid antigen testing for SARS-CoV-2 and influenza A and B in congregate living settings
Source: Front Public Health. 2025 Apr 7;13:1560131. doi: 10.3389/fpubh.2025.1560131 (PMC12009850; doi:10.3389/fpubh.2025.1560131)
Supplement: Supplementary file 1 [file Data_Sheet_1.docx]

**Appendix A**

| **Introduction Preamble**  Hi. My name is [*name of interviewer*] and I’m a [*name of position*] with the Knowledge Translation Program at St. Michael’s Hospital, Unity Health Toronto. Thank you very much for taking part in this interview and taking the time to speak with me today.  *Check in*: Does this time still work for you for the interview? Can hear me clearly and is the volume okay?  Before we get started, I would like to briefly explain the purpose of the project. |
| --- |
| **Objectives**    The goal of this research is to explore the optimal methods for implementing rapid testing to detect COVID-19 and influenza in congregate living settings including shelters, long term care homes (LTCH), and retirement homes (RH) in Ontario. Rapid testing can enable early identification of respiratory infections in residents and staff of congregate living settings. It may reduce risk of transmission of respiratory infections by enabling isolation of infectious individuals. We’re currently conducting interviews with staff and partners at sites implementing rapid testing to understand how the process has been so far. We hope that these conversations will help us determine the feasibility and sustainability of implementing rapid antigen testing in congregate settings in Ontario.  Do you have any questions about any aspects of the project? |
| **Terms of Consent and Confidentiality**  We’ll start by going over the terms of consent in participating in this study. We will then ask you if we have your permission to take part in the study.   - Participation in this interview is completely voluntary. If at any point you wish to stop participating, even after we have started, you are free to do so. - We take the issue of confidentiality seriously. No personal information about you will be shared with anyone outside the study team. - We will be audio taping this interview so that we do not lose any details of our discussion. - Your real name will not appear anywhere in the written transcripts or reports concerning today’s session. All data will remain anonymous. - A summary of the study results will be available if you ask for them once the project is complete. The contact information for study personnel is included in the study information sheet.   Do you have any questions about the terms of consent?  Do I have your consent to participate in this interview?  Do I have your permission to audio tape this interview?  I will not turn on the audio recorder and will ask you a few questions to record your permission.  **Turns on audio recorder**  Today is March 30^th^ 2023 and I’m here with participant F21 to conduct an interview for the evaluation of rapid testing for COVID-19 and influenza in congregate living settings.  **For the purpose of recording your consent to participate, I will ask you again. Do you consent to being interviewed and audio recorded?** |
| **Interview Questions**  **Current Status**   1. What is your role with the site, and what is your role with rapid testing at your site? 2. Have you been administering the RATs, how many? 3. In your perspective, has it been easy to implement rapid testing at your site? Why or why not? *Probe for experiences, challenges* ***[specifically sofia 2 analyzer]*** 4. What has been some major barriers/challenges to implementation been, if any? *Probe for challenges*    1. *If they experienced barriers* – How have you navigated these barriers?   **Feasibility**   1. Do you feel that implementation of rapid testing using the Sofia 2 analyzer is feasible…why or why not? 2. Do you believe you’ve had enough support and resources to implement rapid testing at your site? *Probe for facilitators*    1. What are some forms of support that you think would be helpful moving forward 3. Were there benefits of the differential diagnosis 4. Have you or your team experienced any challenges with implementing rapid testing? *Probe for challenges with residents, personnel, staff absences etc* 5. Is there any alternative to rapid test kits for COVID-19 using the Sofia 2 analyzer    1. What are some factors that facilitate the use of the sofia 2 6. What has worked well with implementing rapid testing? 7. What have been some benefits…. 8. How useful has rapid testing been in the **early detection** of COVID-19 and influenza among staff and residents? *Probe for transmission and outbreaks*   **Sustainability and Scaling up**   1. How do you feel about sustaining rapid testing at your site? 2. *How confident are you about scaling up rapid testing procedures across congregate settings?*   **Perceptions**   1. How was rapid testing perceived by residents? Do you believe additional considerations should be made when implementing?   **Other**   1. Do you have any other comments on any of the topics we’ve discussed today? |
| **Conclusion**  That concludes our interview. Thank you again for taking the time to participate in an interview for the evaluation of rapid testing for COVID-19 and influenza in congregate living settings project. I will now turn off the audio recorder. Thank you. |
