# Supplementary material for: An evaluation of barriers and facilitators to implementing multiplex rapid antigen testing for SARS-CoV-2 and influenza A and B in congregate living settings
Source: Front Public Health. 2025 Apr 7;13:1560131. doi: 10.3389/fpubh.2025.1560131 (PMC12009850; doi:10.3389/fpubh.2025.1560131)
Supplement: Supplementary file 2 [file Data_Sheet_2.docx]

**Appendix B**

**Sofia Multiplex RAT Implementation Post-Interview Brief Demographic Survey**

We want to ask you a few questions as part of a project being conducted at St. Michael's Hospital, Unity Health Toronto to better understand how testing for COVID-19 can improve health and wellbeing for staff and residents in shared living centres.

This questionnaire should take approximately 2 minutes to complete. All of the answers you provide will be completely confidential. There are no major risks to completing this questionnaire. Participation in this questionnaire is voluntary. If a question is not applicable to you or you feel uncomfortable answering it, you may skip the question. Please note that in order to protect your privacy and confidentiality the details you provide will be de-identified prior to analysis and results will only be shared in aggregate form.

This questionnaire is hosted on Qualtrics, which is a secure online data collection system. Results will be stored on Qualtrics’ servers until downloaded onto our secure server for analysis. Qualtrics servers are protected by high-end firewall systems, and scans are performed regularly to ensure that any vulnerabilities are quickly found and patched. Data is stored in a specific Canadian location; it does not float around in the “cloud.” In addition, all data are processed in that location and are not moved to another jurisdictional area (e.g., outside Canada). Access to the data will be limited to the study investigators, study team members. Your data will not be shared with any other researchers or organizations for any reason. If you have reached our participant pool for this study, your information will be deleted immediately and will be notified via email.

Your consent to participate in this questionnaire is demonstrated by your voluntary completion and submission of this questionnaire. If you would like to continue, please press the “Next” button. If you would not like to continue, please close the browser window or press the “End Questionnaire” button.

- **Please enter your first name** __________
- **Please enter your last name** __________
- **What is your current age?**
- 18-25
- 26-30
- 31-35
- 36-40
- 41-45
- 46-50
- 51-55
- 56-60
- 61-65
- 66-70
- 71-75
- 76-80
- 81+
- Prefer not to answer
- **What is your immigration status?**
- Canadian citizen (born in Canada)
- Canadian citizen (foreign born)
- Permanent resident
- Temporary resident
- Prefer not to answer
- Other ___________
- **In our society, people are often described by their race or racial background. These are not based in science, but our race may influence the way we are treated by individuals and institutions and this may affect our health. Which category(ies) best describes you? Check all that apply:**
- Black (e.g. Ghanaian, Kenyan, Somali, Jamaican, Canadian, American)
- East Asian (e.g. Chinese, Japanese, Korean, etc.)
- Indigenous (e.g. First Nations, Métis, Inuk/Inuit)
  - Do you identify as Two-Spirit? Yes/No
- Latinx (e.g. Salvadorian, Argentinean, Chilean, Mexican)
- Middle Eastern (e.g. Egyptian, Lebanese, Iranian)
- South Asian (e.g. Indian, Pakistani, Sri Lankan etc)
- Southeast Asian (e.g. Filipiono, Cambodian, Vietnamese, Thai etc.)
- White (e.g. English, Italian, Russian, Canadian, American)
- Other: ________________
- Prefer not to answer
- **What was your sex assigned at birth? *(check one)***
- Female
- Male
- Intersex
- Prefer not to answer
- **What is your current gender identity? *(check one)***
- Woman
- Man
- Transgender
- Gender fluid or Gender non-binary
- Two-Spirit (Indigenous)
- Another (Specify) __________
- Prefer not to answer
- **What is the highest level of education you have completed?**
- 12^th^ grade or less
- Graduated high school or equivalent
- Some college/university, no degree
- College/University degree
- Post-graduate degree
- **What is your current employment status?**

1. Full-time
2. Part-time

- **What is your current role?** __________
- **How many years have you worked in this role?** __________

1. Less than a year
2. 1-3 years
3. 4-6 years
4. 7-10 years
5. >10 years

- **How many years have you worked at the site where you are currently employed?**

1. Less than a year
2. 1-3 years
3. 4-6 years
4. 7-10 years
5. >10 years

- **What is your level of involvement in facilitating rapid testing at your site (in either a management/coordination/front-line capacity)?**

1. Limited involvement
2. Moderate involvement
3. High level of involvement
